# The Anatomy of Rape

**Published:** 1897-02

**Authors:** 


### THE ANATOMY OF RAPE.

In an interesting article on the Anatomy of Rape which appeared in the *University Medical Magazine*, by Dr. W. Constantine Goodell, the following is a brief summary:

1. That complete penetration, with the ejaculation of semen, is not necessary alone to constitute rape, but that merely partial penetration of the male organ between the lips of the vulvar orifice, with or without ejaculation, is sufficient.
2. That defloration of a very young virgin, even supposing that force was not used, should show evidences of rape, from the very disproportion in size between the penis and the vagina.
3. That the hymen is of the greatest value in determining whether coitus has taken place or not, notwithstanding the unanimous opinion of the older writers to the contrary.
4. That there is a line of demarcation, formed by the edge of the genital mucous membrane, *within* which, impinging of the male organ, with or without the ejaculation of semen, would as surely constitute rape as though complete penetration had occurred.
5. The great difficulty and often absolute impossibility of determining whether rape has been committed, when only partial penetration has been secured and ejaculation has not taken place.

6. The inability to successfully differentiate between marks of violence due to the penis and abrasions from attrition caused by walking, or some form of traumatism.

